# A Study of Patient Preferences for the Treatment of Non–small Cell Lung Cancer in Western China: A Discrete-Choice Experiment

**DOI:** 10.3389/fpubh.2021.653450

**Published:** 2021-03-26

**Authors:** Fei Liu, Haiyao Hu, Jing Wang, Yingyao Chen, Sun Hui, Ming Hu

**Affiliations:** ^1^West China School of Pharmacy, Sichuan University, Chengdu, China; ^2^West China School of Medicine, West China Hospital, Sichuan University, Chengdu, China; ^3^School of Public Health, Fudan University, Shanghai, China

**Keywords:** discrete-choice experiment, non-small cell lung cancer, patient preference, evidenced-based, mixed logit model

## Abstract

**Background:** Non–small cell lung cancer (NSCLC) is the most common histologic type of lung cancer, accounting for 70–85% of all lung cancers. It has brought a heavy burden of disease and financial cost to families, society, and the nation of China. Patients have differing preferences for treatment because of their varying physical conditions and socioeconomic backgrounds, which ultimately affects the choice of treatment as well as treatment outcomes. For better and sustained health outcomes, it is vital to understand patients' preferences. We can then provide medical services to match these preferences and needs rather than basing treatment on our clinical viewpoints alone.

**Objectives:** The aim of this study was to elicit patient preferences for treatment using a discrete-choice experiment and to explore the value/importance that patients place on the different attributes of treatment in order to provide a basis for clinical decision making and patient health management.

**Methods:** The study was conducted with NSCLC patients from three typical hospitals in southwestern China. After identifying patient-relevant treatment attributes via literature review and qualitative semi structured interviews, a discrete-choice experiment (DCE) including seven patient-relevant attributes was conducted using a fractional factorial SAS design. The empiric data analyses of patients were performed using mixed logit models.

**Results:** NSCLC patients (*N* = 202) completed a survey via a face-to-face interview. Among the seven attributes, the following were considered important: progression-free survival, disease control rate, cost, weakness/fatigue, and nausea/vomiting; mode of administration and rash were considered less important. A clear preference for an increase in progression-free survival and disease control rate was demonstrated. Compared with 5 months of progression-free survival, respondents were willing to pay more (19,860 RMB) for 11 months of progression-free survival (coef.: 0.687). Compared with a 60% rate of disease control, respondents were willing to pay more (19,940 RMB) for a 90% rate of disease control (coef.: 0.690).

**Conclusions:** This study demonstrates the value of DCEs in determining patient preferences for the treatment of NSCLC. The results indicate that not only efficacy factors (such as progression-free survival and disease control rate) were considered but also other factors (such as side effects and treatment costs) and trade-offs between attributes were held to be important. These results are in accord with expectations and can provide evidence for more effective and efficient treatment results. Furthermore, the current results can increase benefits if the presented therapies can be designed, assessed, and chosen based on patient-oriented findings.

## Background

In 2018, some 9.6 million people died of cancer worldwide; of these, 2.09 million died of lung cancer (1). Non–small cell lung cancer (NSCLC) is the most common histologic type of lung cancer, accounting for 70–85% of all lung cancer (2). Advanced patients face the challenge of high mortality and low survival rates; only 15% of these patients live as long as 5 years after diagnosis (3, 4). NSCLC brings a heavy disease and economic burden to families, societies, and the nation of China. In 2016, Migliorino et al. estimated that the overall health care cost in Italy over 16.4 months of observation was 25,859 euros per patient (5). In China, lung cancer takes first place in terms of incidence and mortality, and NSCLC has also brought a heavy economic burden to Chinese families (6, 7). Ding's calculations show that the average cost of hospitalization for a patient with NSCLC in a tertiary hospital in China is 26,958 RMB (8). With the development of targeted therapy over the past decade, the emergence of epidermal growth factor receptor tyrosine kinase inhibitors (EGFR-TKIs) has brought important breakthroughs to the treatment of advanced NSCLC. According to the National Comprehensive Cancer Network (NCCN) Clinical Practice Guideline in Oncology: Non–Small Cell Lung Cancer, version I 2015 (9, 10), the diagnosis and treatment of advanced NSCLC emphasize tumor molecular detection and targeted therapy guided by molecular detection. EGFR-TKIs have become the standard first-line treatments for advanced NSCLC with an *EGFR* mutation or *ALK* gene fusion. However, the high cost of targeted drugs deters many patients. Bie et al. calculated that the monthly cost of targeted therapy was about 12,317–19,062 RMB (11). Patients' preferences may vary owing to their different physical conditions and socioeconomic backgrounds; these ultimately affect the choice of treatment and its effects (12). Given that the assessment and choice of medical resources performed by patients and medical service providers are not identical, simply evaluating the therapeutic effect from the perspective of the medical service provider may not meet the actual needs of patients. This is why it is important to consider patient choice when determining a course of treatment. It is not only the key to improve compliance of patients, but also an important way to improve the health service utility and optimize resource allocation under the current patient-centered medical model.

As an attribute-based measure of benefit, discrete choice experiments (DCEs) have been increasingly utilized in health economics to examine the importance of an attribute in health care delivery, with consideration given to both to the patient experience and health outcomes, as well as to trade-offs between these and willingness to pay (WTP) for different attributes (13). DCEs are a stated-preference method that involves the generation and analysis of choice data and the creation of hypothetical markets that can be constructed to suit relevant research questions (14). These types of studies feature a high degree of realism and are easy for patients to handle (15). Typically, DCEs are implemented as surveys in which respondents are presented with several choice sets, each containing several alternatives between which respondents are asked to choose (16). The dependent variable in a patient preference study is choice, while the independent variables are the attributes and levels characterizing the alternatives. Econometric analysis yields estimated preference parameters, which represent the influence of specific attributes or attribute levels on choices (17). As an effective method to quantify patient preferences for different treatments, discrete choice experiments are practical within the context of many different cultures.

Researchers outside of China have adopted discrete choice experiments to demonstrate the treatment preference of patients with malignant tumors. Studies conducted within China, however, have primarily focused on exploring information demand in patients with malignant tumors. Very few studies have been conducted on how to choose anti-cancer treatment for patients before accepting therapy, and no academic research has been conducted on the treatment preferences of patients with non-small cell lung cancer (NSCLC). This study, which utilized a discrete choice experiment to explore the main factors, as well as the degree of influence of each factor, on treatment preference from the perspective of NSCLC patients, was conducted to provide policy and decision-making advice for relevant decision-making departments within the government.

## Methods

### Discrete-Choice Experimental Methodology

A discrete-choice experiment (DCE) survey was developed following good research practices for stated-preference studies as outlined by a report of the ISPOR Conjoint Analysis Good Research Practices Task Force (18). This method is based on the assumption that decisions can be described by a number of key attributes and that an individual's choice is influenced by the levels of these attributes (19). Each respondent has to make trade-offs between attributes and the levels of said attributes (15).

#### Identifying the Attributes of Treatment

To select attributes for this study, “non-small cell lung cancer,” “main therapeutic drugs,” “main efficacy indicators,” “main safety indicators,” “pharmacoeconomics indicators,” “preference research,” and “influencing factors” were used to search the official website of WHO, CNKI, CBM and other Chinese journal literature databases as well as Elsevier, EMBASE, PubMed and MEDLINE (time range from literature reports to June 2017). According to the results of literature research, a semi-structured interview, which was then utilized in conducting qualitative interviews with NSCLC patients, was drawn up among the relevant physicians and experts to clarify the exact meaning of each attribute and level.

#### Assigning Levels to the Attributes

Based on the results of the aforementioned literature review and semi-structured interviews, seven attributes of treatment preference for patients with NSCLC were determined. Among these, treatment cost was a continuous variable, and disease progression-free survival, control rate, rash, nausea/vomiting, weakness/fatigue, and mode of administration were categorical variables. Six attributes were assigned three levels, while the mode of administration attribute was assigned two levels. A list of these attributes and levels is presented in Table 1.

**Table 1:** Attributes and levels for the choice tasks.

**Attributes**	**Description to patients**	**Levels and Level description**		**Abridged general view**
Progression-free survival	It refers to the time from the start of treatment until the tumor appears to be further deteriorated; this study fixes the overall survival and gives a choice of progression-free survival over time.	High Medium Low	11 months 8 months 5 months	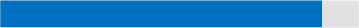 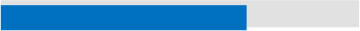 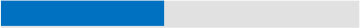
Disease control rate	The number of cases with remission and stable lesions after treatment accounted for the number of cases that could be evaluated.	High Medium Low	90% 75% 60%	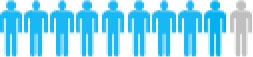 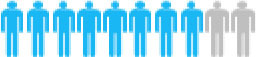 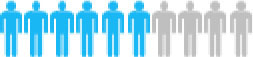
Rash	Treatments can cause a varying degrees rash for as long as you take the treatment.	None Mild Moderate	No rash <1/10 of the body more than 1/3 of the body	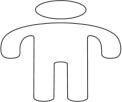 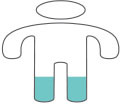 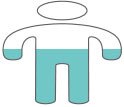
Nausea and vomiting	Treatments can cause nausea and vomiting as long as you take the treatment.	Mild Moderate Severe	1 time in 24 h 2–5 times in 24 h more than 6 times in 24 h	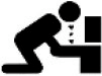 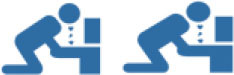 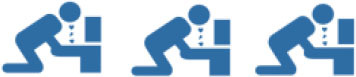
Weakness and fatigue	When you take the treatment you may feel even weaker or more tired.	Mild Moderate Severe	It is difficult to do physical exercises such as climbing stairs or running. Can't work and take care of yourself. You need to remain in bed	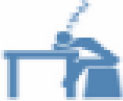 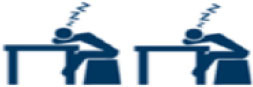 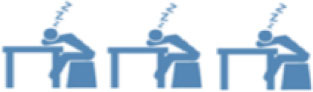
Cost	How much will you pay for the treatment.	High Medium Low	5 ten thousand per months 2.5 ten thousand per months 1 ten thousand per months	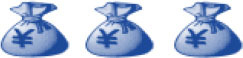 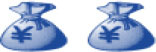 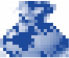
Mode of administration	In general, treatments can be administered by infusion or taken as pills.	Infusion Oral		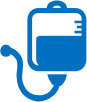 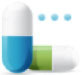

### Study Participants

According to geographical location, level of economic development, and the particular attributes of respondents, sampling was conducted using judgment sampling. NSCLC patients from three tertiary hospitals in Chengdu, which is the most concentrated city of patients with lung cancer in Western China, were chosen as the survey subjects: West China Hospital, Sichuan University, Sichuan Provincial People's Hospital, and Sichuan Cancer Hospital.

Patients were eligible for this study if they (1) were 18 years of age or over, (2) had a confirmed diagnosis of NSCLC, (3) had received chemotherapy in the past (including both outpatient and inpatient treatment), and (4) had provided informed consent. Patients were excluded for this study if they (1) had been diagnosed with mental illness or were unable to cooperate with the survey, (2) had received only drug donation treatment (patients who received partial drug donation were not excluded), or (3) did not complete the questionnaire.

According to Orme's calculation, the minimal sample size recommended for this study was at least 84 (20). Considering the need for subgroup analysis of disease severity, patient gender, age, economic status, health insurance status, and so on, a sample size of 300 NSCLC patients was required to guarantee statistically robust estimates (16). The survey was administered on September 29, 2017. Judgment sampling was conducted according to geographic location, level of economic development, and the particular attributes of the respondents. The questionnaire's sample size and sampling distribution are shown in Table 2.

**Table 2:** Patients' preferences study on treatment of non-small cell lung cancer–collection of the questionnaires.

**Institution**	**Patient questionnaire**	**Effective questionnaire**	**Effective rate**
West China Hospital,Sichuan University	100	77	77.0%
Sichuan Provincial People's Hospital	150	104	69.3%
Sichuan Cancer Hospital	50	21	42.0%
Total	300	202	67.3%

Ethical approval for the patient preference study was granted by the Human Research Ethics Committee, Fudan University, Shanghai, China, approval number IRB#2017-09-0638.

#### Choice of Hypothetical Treatment Models

The combination of attributes and attribute levels (six attributes with three levels and one attribute with two levels) resulted in 3^6^ × 2^1^ = 1,458 potential hypothetical treatment models and 1,062,153 choice pairs. In order to develop the minimal number of necessary choice pairs that would allow for the estimation of all main effects, a fractional factorial design was utilized (17, 21). Finally, a D-efficient, main-effects experimental design alternative set with 18 choice pairs was developed using SAS version 9.3 software. In this alternative set, each respondent would make choices in 18 different choice tasks. An example of a choice task is presented in Table 3.

**Table 3:** Example choice set of the discrete-choice experiment.

**1**	**A treatment**		**B treatment**	
Progression-free survival	8 months	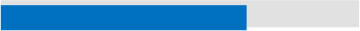	5 months	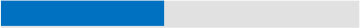
Disease control rate	60%	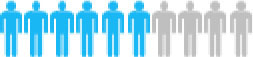	75%	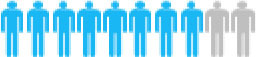
Rash	<1/10 of the body	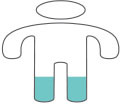	No rash	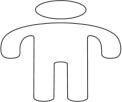
Nausea and vomiting	1 time in 24 h	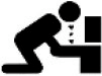	2–5 times in 24 h	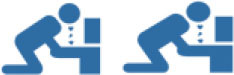
Weakness and fatigue	Can't work and take care of yourself.	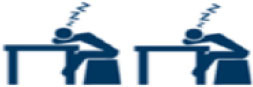	It is difficult to do physical exercises such as climbing stairs or running.	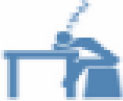
Cost	5 ten thousand per months	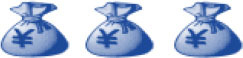	2.5 ten thousand per months	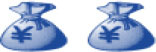
Mode of administration	Infusion	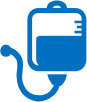	Oral	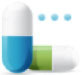
Which choice do you prefer?				

#### Development of Questionnaire and Data Collection

The DCE questionnaire commenced with a detailed written description of each attribute and its levels, followed by a set of DCE questionnaires. Questions regarding sociodemographic characteristics, demographic information (e.g., age, gender, marital status, living situation, and smoking habit), and the patient's experience with NSCLC (e.g., time since diagnosis, cancer type, cancer stage, and past experiences with therapy) were asked.

The DCE scenarios generated the patient preferences by presenting respondents with a series of hypothetical treatment scenarios while systematically varying attribute levels based on an experimental design. In our study, patients were presented with hypothetical treatment models described in terms of the treatment attributes and asked to choose from two hypothetical models, treatment A and treatment B.

A pilot test that involved investigating 30 NSCLC patients from West China Hospital was conducted to modify the questionnaire (The data from these 30 patients were not included in the data analysis of the formal investigation). Oncologists from the West China Hospital were consulted to evaluate the quality of the questionnaire.

### Data Analysis

#### Statistical Model

DCEs are mainly based on the random utility theoretic (RUT) framework. Under this framework, an individual respondent is assumed to choose the alternative for which he or she holds the highest utility (20). Models were estimated using Stata software version 14.0; we made use of a mixed logit model (MXL) (16, 21).

The utility *U*_*n,j,t*_ patients *n* derive from a particular choice of treatment, *j* ∈ {1, 2, ⋯ , *J*} in the choice task set *t* consists of two parts:

(1) $$\begin{array}{l} U_{n,j,t}=A_{n,j,t}+\varepsilon_{n,j,t} \end{array}$$

where *A*_*n,j,t*_ is the observable partial utility, and ε_*n,j,t*_ is the unobservable partial utility. Besides, *A*_*n,j,t*_ can be expressed as the linear combination of the *m* observable treatments $\text{T}={\left[T_{1},T_{2},\cdots \;,T_{m}\right]}^{T}$ with weight vector β, i.e.,

$$\begin{array}{l} A_{n,j,t}=f\left({\text{T}}_{n,j,t}\right)=\beta_{0}+\beta^{T}{\text{T}}_{n,j,t}=\beta_{0}+\beta_{1}T_{1,n,j,t}+\beta_{2}T_{2,n,j,t}\\ \;+\cdots +\beta_{m}A_{m,n,j,t} \end{array}$$

Each attribute—such as progression-free survival and disease control rate has its corresponding weight β_0_, β_1_, ⋯ , β_*m*_. $\varepsilon_{n,j,t}\sim N\left(0,\sigma^{2}\right)$ is a random effect of observable factors on utility, which can be assumed as a function of treatment alternatives and individual preference differences.

The probability *P*_*n,j,t*_ of patient *n* choosing treatment *i* in the choice task set *t* to obtain the maximum utility *U*_*n,j,t*_ can be shown as Equation (2):

(2) $$\begin{array}{l} P_{n,j,t}=\mathbb{P}\left[U_{n,i,t}>U_{n,j,t}\right]\\ \;=\mathbb{P}\left[A_{n,i,t}+\varepsilon_{n,i,t}>A_{n,j,t}+\varepsilon_{n,j,t}\right]\\ \;=\mathbb{P}\left[A_{n,i,t}-A_{n,j,t}>\varepsilon_{n,i,t}-\varepsilon_{n,j,t}\right],\;\forall j\neq i \end{array}$$

If the random term ε_*n, i, t*_ obeys the “independence between unrelated options,” a multinomial logit model can be applied. Otherwise, a nested logit model or a mixed logit model can be used to estimate the parameters in the above equation.

#### Statistical Analysis

A mixed logit model was used to analyze DCE data. Except that the cost (copayment) is specified as a continuous variable in all models, other attribute variables are encoded as virtual variables. All the coefficients of the model are assumed to be of normal distribution.

The model regression coefficient (β_0_−β_*m*_) reflects the nature of the treatment plan and the direction and size of the choice of treatment options. Positive regression coefficients indicate that this attribute has a positive effect on the preference (e.g., the longer the progression-free survival, the more likely the patient to choose the treatment). On the contrary, there is a negative effect (for example, the greater the side effects, the more likely the patient not to choose the treatment). The absolute value of the regression coefficient reflects the influence of the attribute on the choice intention of treatment. When the monetary attributes of the treatment cost (copayment) were included in the study, the monetary value of the nonmonetary attributes of the treatment could be estimated as a negative ratio of the regression coefficient of the nonmonetary attributes to the regression coefficient of the monetary attributes of the treatment cost (copayment). Thus, DCEs can be used to analyze the patient's willingness to pay to change the level of an attribute—that is, the monetary value of the level of an attribute. A positive sign indicates that the patient is willing to pay more for the attribute, whereas a negative sign indicates compensation for the patient's ability to accept the attribute. Assuming that *T*_1_ represents the cost of treatment, the monetary value of the treatment attribute *T*_*m*_ can be expressed as Equation 3:

(3) $$\begin{array}{l} \text{WTP}\left(T_{m}\right)=\frac{\frac{\partial U}{\partial T_{m}}}{\frac{\partial U}{\partial T_{1}}}=\frac{\beta_{m}}{\beta_{1}} \end{array}$$

Cost was the only variable that could logically be coded as a continuous variable. Levels of efficacy, side effects, and mode of administration are descriptive and thus categorical. One level of each attribute was chosen as a reference level (omitted level). We utilized dummy-variable coding to allow for a parameter to be estimated for remaining levels. In the dummy-variable coding, each nonomitted attribute level is assigned a value of “1” when the level is present in the corresponding profile and “0” when another nonomitted level is present in the corresponding profile (22). However, unlike effects coding, all nonomitted levels are coded as “0” when the omitted level is present. To account for clustering and preference heterogeneity, a mixed logit model was estimated using Stata 14.0.

The parameters of the choice model for each attribute level can be expressed as regression coefficients. Positive parameter estimates indicate a positive utility and are associated with a regression coefficient greater than zero, as a positive utility would be associated with a preferred choice. Otherwise, there is a reverse effect. The magnitude of the absolute value of the regression coefficient reflects the extent to which the attribute influences treatment choice intention.

An alternative approach to evaluating importance is to measure the willingness of respondents to make trade-offs. This methodology is consistent with welfare economics (14, 23, 24). As we included cost as an attribute, we were able to estimate willingness to pay. Within the context of treatment profiles, inclusion of a price proxy (such as cost) allows the researcher to estimate the monetary value of attributes of a treatment. In other words, the researcher is able to determine how much a respondent would be willing to give up in order to obtain an improvement in other aspects of the treatment. This can be estimated as the ratio of the value of the coefficient of interest to the negative of the cost attribute (25).

## Results

Of the 300 questionnaires sent out to the patients, 202 usable questionnaires were returned, giving a response rate of 67.3%. Table 4 reports the social demographic characteristics of the respondents. Of the 202 respondents, 79 were female and 123 were male. The average age of the respondents was 58.6 years with a range of 35–79 years. The majority of respondents was male (60.9%), married (95.5%), unemployed (83.2%), smokers (57.4%), and lived with a spouse (64.4%). The largest number of respondents self-reported having been diagnosed with NSCLC <6 months earlier (45.5%), whereas 36.6% were diagnosed between 6 and 12 months and 17.9% more than a year earlier. The majority of respondents reported an adenocarcinoma NSCLC histology (67.3%) and had late-stage disease, with 34.6% reporting stage III and 39.6% reporting stage IV.

**Table 4:** Demographics and baseline characteristics of the sample.

**Sample characteristic**	**Subjects**	**Percentage**
	***N* = 202**	**(%)**
**Gender - no**.		
Male	123	60.9%
Female	79	39.1%
**Age – years**		
55 years and younger	83	41.1%
55–65 years	60	29.7%
65 years and older	59	29.2%
Mean	58.6	
**Economic level - RMB**		
50,000 RMB and less	100	49.5%
More than 50,000 RMB	76	37.6%
Unknown	26	12.9%
**Marital Status - no**.		
Married	193	95.5%
Not married	1	0.5%
Divorced	3	1.5%
Windowed	5	2.5%
**Education level - no**.		
Primary school and below.	40	19.8%
Junior school certificate	66	32.7%
High school certificate	31	15.3%
Vocational school	13	6.4%
Junior college	24	11.9%
Bachelor degree	27	13.4%
Master degree or higher	1	0.5%
**Employment status – no**.		
Employed full-time	16	7.9%
Self-employed	18	8.9%
Unemployed	46	22.8%
Retired	84	41.6%
Unable to work due to cancer	38	18.8%
**Smoking habit - no**.		
No	86	42.6%
Yes	116	57.4%
**Living situation – no**.		
Live alone	5	2.5%
Live with children	24	11.9%
Live with spouse	130	64.4%
Live with children and spouse	41	20.3%
Others	2	1.0%
**Time since diagnosis – no**.		
<6 months	92	45.5%
6 months−1 year	74	36.6%
1–2 years	22	10.9%
2–5 years	10	5.0%
More than 5 years	4	2.0%
**Cancer type – no**.		
Adenocarcinoma	136	67.3%
Squamous	61	30.2%
Adenocarcinoma and squamous	2	1.0%
Large cell carcinoma	0	0.0%
Others	3	1.5%
**Cancer stage – no**.		
Stage Ia, Ib (non-advanced)	14	6.9%
Stage IIa, IIb (non-advanced)	38	18.8%
Stage IIIa (non-advanced)	39	19.3%
Stage IIIb (advanced)	31	15.3%
Stage IV (advanced)	80	39.6%

As shown in Table 5, when patients were choosing an NSCLC treatment, the following factors affected their decisions: progression-free survival, disease control rate, nausea/vomiting, weakness/fatigue, and cost. The more important factors were progression-free survival and disease control rate, followed by weakness/fatigue, nausea/vomiting, and cost. Patients were more inclined to prefer a longer lasting progression-free survival, higher disease control rate, fewer instances of nausea/vomiting or weakness/fatigue, and less cost for treatment, whereas they showed no preference for mode of administration or rashes.

**Table 5:**
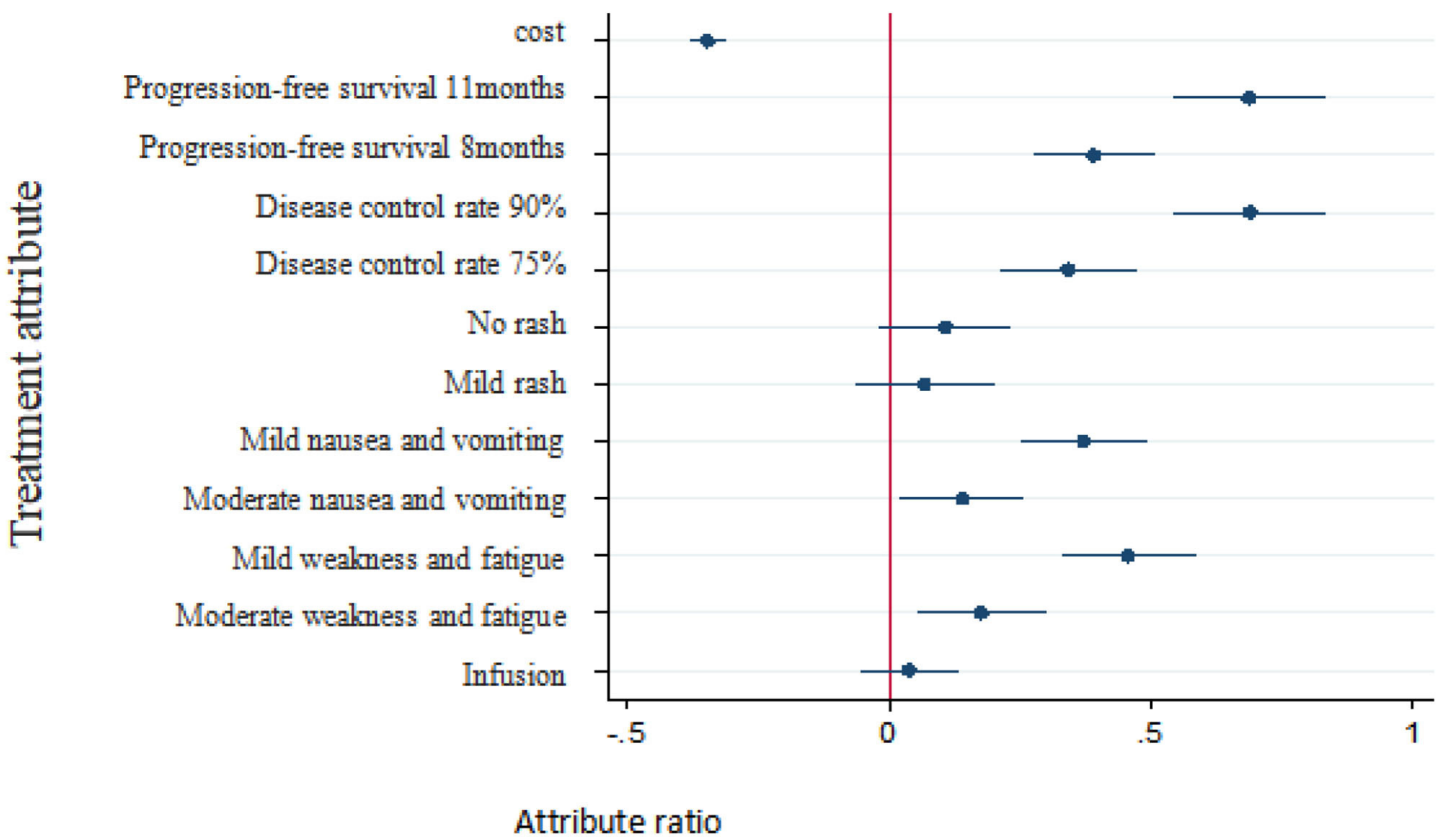
Preference of treatment attributes and levels for patients with non-small cell lung cancer.

The monetary value of each nonmonetary attribute of treatment can be estimated as the negative of the ratio of the nonmonetary attributes regression coefficient to the monetary attributes cost regression coefficient. Therefore, willingness to pay for changes in the attribute level of a certain treatment can be analyzed by discrete-choice experiments. The plus sign indicates a patient's willingness to pay for obtaining the attribute level and the minus sign indicates his or her willingness to compensate for accepting the attribute level. The regression results of patients' preferences for treatment as well as their estimated willingness to pay and 95% confidence intervals are shown in Table 6.

**Table 6:** Regression results and WTP.

**Attributes of treatment**	**β**	**Coefficient (Standard error)**	**WTP (ten thousand) (95% CI)**
Cost	β1	−0.346^***^ (0.0173)	–
Progression-free survival 11 months (Reference level: 5 months)	β2	0.687^***^ (0.0748)	1.986 (1.560; 2.412)
Progression-free survival 8 months (Reference level: 5 months)	β3	0.391^***^ (0.0590)	1.129 (0.789; 1.470)
Disease control rate 90% (Reference level: 60%)	β4	0.690^***^ (0.0744)	1.994 (1.583; 2.404)
Disease control rate 75% (Reference level: 60%)	β5	0.341^***^ (0.0668)	0.985 (0.609; 1.362)
No rash (Reference level: Moderate rash)	β6	0.107 (0.0644)	0.310 (−0.059; 0.680)
Mild rash (Reference level: Moderate rash)	β7	0.068 (0.0679)	0.198 (−0.192; 0.588)
Mild nausea and vomiting (Reference level: Severe nausea and vomiting)	β8	0.372^***^ (0.0625)	1.075 (0.693; 1.457)
Moderate nausea and vomiting (Reference level: Severe nausea and vomiting)	β9	0.139^*^ (0.0604)	0.402 (0.051; 0.754)
Mild Weakness and fatigue (Reference level: Severe Weakness and fatigue)	β10	0.457^***^ (0.0656)	1.320 (0.918; 1.722)
Moderate Weakness and fatigue (Reference level: Severe Weakness and fatigue)	β11	0.175^**^ (0.0627)	0.507 (0.143; 0.872)
Infusion (Reference level: Oral)	β12	0.0381 (0.0476)	0.110 (−0.159; 0.380)
Constant term	β13	0.0903 (0.0503)	–
Sample size		202	
Observation value		7,272	
log likelihood function		−2,050.0408	

WTP presented in ten thousand RMB per month; 95% confidence intervals in parentheses.

* Significant at 5% level,

** significant at 10% level,

*** significant at 1% level.

*Chi-square Test: χ^2^ = 52.78, P = 0.0000*.

The results showed that, compared with the reference level, respondents were willing to pay 19,860 RMB per month for 11 months of progression-free survival and were willing to pay 19,990 RMB per month for a 90% disease control rate. Additionally, in exchange for milder weakness and fatigue, respondents were willing to pay 13,200 RMB per month, and in exchange for milder nausea/vomiting, 10,755 RMB per month.

The changes in simulation results of patient demand probability after improving treatment are shown in Table 7. It can be seen from the results that the improvement of the treatment plan had an important influence on the choice preference of stakeholders.

**Table 7:** Simulated preferences for treatment under various potential treatment scenarios.

**Attributes and reference level**	**The change in the probability**	**95% Confidence intervals**
Cost: 5 ten thousand (ref: 1 ten thousand)	−59.91%^***^	(−64.26%; −55.57%)
Cost: 2.5 ten thousand (ref: 1 ten thousand)	−25.37%^***^	(−27.75%; −23.00%)
Progression-free survival: 11 months (ref: 5 months)	33.06%^***^	(26.53%; 39.60%)
Progression-free survival: 8 months (ref: 5 months)	19.29%^***^	(13.72%; 24.86%)
Disease control rate: 90% (ref: 60%)	33.17%^***^	(26.69%; 39.66%)
Disease control rate: 75% (ref: 60%)	16.88%^***^	(10.52%; 23.24%)
Rash: none (ref: moderate)	5.36%	(−0.93%; 11.65%)
Rash: mild (ref: moderate)	3.42%	(−3.22%; 10.06%)
Nausea and vomiting: mild (ref: severe)	18.38%^***^	(12.47%; 24.30%)
Nausea and vomiting: moderate (ref: severe)	6.95%^*^	(1.06%; 12.84%)
Weakness and fatigue: mild (ref: severe)	22.44%^***^	(16.34%; 28.55%)
Weakness and fatigue: moderate (ref: severe)	8.75%^**^	(2.66%; 14.85%)
Mode of administration: Infusion (ref: Oral)	1.91%	(−2.75%; 6.57%)

* Significant at 5% level,

** significant at 10% level,

*** *significant at 1% level*.

Probability changes with patient needs compared with the reference level (i.e., baseline): (1) With treatment cost increased to 50,000 RMB per month, the probability of choosing the treatment was reduced by 59.91%. With treatment cost increased to 25,000 RMB per month, the probability of choosing the treatment was reduced by 25.37%. (2) With progression-free survival increased to 11 months, the probability of choosing this treatment program increased by 33.06%. With the progression-free survival increased to 8 months, the probability of choosing the treatment increased by 19.29%. (3) With disease control rates increased to 90%, the probability of choosing the treatment increased by 33.17%. With disease control rates increased to 75%, the probability of choosing the treatment increased by 16.88%. (4) With mild nausea/vomiting, the probability of choosing the treatment increased by 18.38%. With moderate nausea/vomiting, the probability of choosing the treatment increased by 6.95%. (5) With mild weakness and fatigue, the probability of choosing the treatment increased by 22.44%. With moderate weakness and fatigue, the probability of choosing the treatment increased by 8.75%. No statistically significant change in the probability of rash or mode of administration was observed.

## Discussion

At present, discrete-choice experiments have been adopted to demonstrate the treatment preferences of patients with malignant tumors as well as other conditions such as diabetes and low back pain. They have also been used in Community Pharmacy Asthma Services (24). However, few studies have been conducted on how patients in China would choose an anticancer treatment prior to beginning therapy. Our study was based on a sample of patients residing in western China. Analysis of treatment preferences of patients with NSCLC showed that patients prefer treatment with longer progression-free survival, higher disease control rates, fewer side effects (e.g., nausea/vomiting and weakness/fatigue), and lower treatment costs, while rash and mode of administration were basically irrelevant to them. These results are in line with the expectations of treatment based on commonality and total experience (26).

In 2012, Bridges explored the trade-off between the attributes of treatment options for patients with advanced NSCLC in the United Kingdom (27). Mühlbacher et al. studied the treatment preferences of German NSCLC patients and found that progression-free survival and cancer-related symptoms had important effects on treatment decisions (15). Our study observed similar results that progression-free survival was most important for patients and mode of administration was less important, which is not consistent with expectations. The results show that patients had a tendency to choose infusion, which may have been an artifact related to the population of patients being investigated. Specifically, Chinese patients are accustomed to receiving infusion therapy. Thus, the cultural differences and medication habits over the past 20 years may bring about the difference of preference. The results of willingness to pay shows that respondents were willing to pay more for higher disease control rates and longer progression-free survival. Compare to the reference level 5 months, patients were willing to pay 19,860 RMB a month for 11 months of progression-free survival. In the evaluation of pharmacoeconomics, referring to China's per capita GDP of three times in 2018, the acceptable cost of a QALY is about 193,800 yuan, which is 16,150 yuan converted to 1 month of healthy life. The reason of the WTP results of patients' perspective evaluation were higher may be the DCE was based on the assumption scenarios and the payment level was determined subjectively by patients, and Chengdu is a big city with relatively higher income compared to the national average, may also leading to higher acceptable cost of a QALY. Besides, the framework of the DCE questionnaire, especially costs, which depends on the policy of reimbursement of each countries, and options, will affect the results of WTP (25, 28, 29).

Besides these attributes, there are other factors such as gender, age, economic level, education level, working status, smoking history and tumor stage may have an impact on the treatment options of patients with NSCLC, especially for WTP. For example, our study showed that compared with the reference level, in order to obtain 90% disease control rate, the patients with higher economic level are willing to pay about 26,200 RMB more per month than the patients with lower economic level. The same goes for the factor of education level.

## Conclusion

Our study found that important factors affecting the treatment choice for patients with NSCLC included primarily progression-free survival and disease control rate. Secondary factors were cost, nausea/vomiting, and weakness/fatigue, whereas rash and mode of administration were relatively unimportant. A focus on progression-free survival and disease control rate will greatly increase the choice probability of patients with NSCLC, and improvements in cost will significantly reduce the choice probability of patients with NSCLC.

Based on a theoretical analysis of DCEs, this study quantitatively analyzed the treatment preferences of patients with NSCLC via a discrete-choice model. This model avoids the dilemma of the choice of treatment in actual clinical practice and solves an issue with traditional patient preference analysis methods, which are unable to quantitatively analyze the treatment of NSCLC patients with the attributes of each treatment. Our paper reveals how Chinese lung cancer patients evaluate different aspects of drug therapy and their outcomes. Therefore, these data can be used to combine patient evidence with clinical evidence, thus filling a gap in existing knowledge. Furthermore, incorporating patient perspectives into treatment and reimbursement decisions can optimize the allocation of scarce resources.

## Limitations

There are some limitations to our study. First, the seven most important attributes may not reflect the effects of other attributes on preferences. Second, although treatment cost was included in the treatment attributes to quantify the monetary value of nonmonetary attributes, there is still controversy about the appropriateness of willingness to pay estimates in using mixed logit models based on discrete selection experimental data. It will be necessary to discuss and verify the inclusion of monetary attributes in the future. Third, there is no standard sample size for conducting DCEs at present; therefore, extending the research results to the general population of patients with NSCLC may not be appropriate.

## Data Availability Statement

The original contributions presented in the study are included in the article/supplementary material, further inquiries can be directed to the corresponding author/s.

## Ethics Statement

The studies involving human participants were reviewed and approved by Key lab of Health Technology Assessment, Ministry of Health (Fudan University). Written informed consent for participation was not required for this study in accordance with the national legislation and the institutional requirements.

## Author Contributions

MH and YC conceived and designed the research. FL, HH, JW, and SH collected data and conducted the research. FL and HH wrote the initial paper and had primary responsibility for final content. MH revised the paper. All authors contributed to the article and approved the submitted version.

## Conflict of Interest

The authors declare that the research was conducted in the absence of any commercial or financial relationships that could be construed as a potential conflict of interest.
